# Perceived and Objective Fertility Risk Among Female Survivors of Adolescent and Young Adult Cancer

**DOI:** 10.1001/jamanetworkopen.2023.37245

**Published:** 2023-10-11

**Authors:** Hena Naz Din, Savitri Singh-Carlson, Heather L. Corliss, Sheri J. Hartman, David Strong, Hala Madanat, H. Irene Su

**Affiliations:** 1 Herbert Wertheim School of Public Health and Human Longevity Science, University of California San Diego, La Jolla; 2 School of Public Health, San Diego State University, San Diego, California; 3 School of Nursing, San Diego State University, San Diego, California; 4 Center for Research on Sexuality and Sexual Health, San Diego State University, San Diego, California; 5 Institute for Behavioral and Community Health, San Diego State University, San Diego, California; 6 Moores Cancer Center, University of California San Diego, La Jolla; 7 Division of Research and Innovation, San Diego State University, San Diego, California; 8 Institute for Behavioral and Community Health, San Diego State University, San Diego, California; 9 Division of Reproductive Endocrinology and Infertility, University of California San Diego, La Jolla

## Abstract

**Importance:**

Fertility is important to many survivors of adolescent and young adult (AYA) cancer, yet data on this population’s fertility perceptions and their alignment with objective infertility risk are scant.

**Objective:**

To assess whether estimated treatment gonadotoxicity and posttreatment menstrual pattern are associated with higher infertility risk perception.

**Design, Setting, and Participants:**

This retrospective cohort study included female young adult survivors of cancer diagnosed between ages 15 and 39 years were recruited between March 25, 2015, and September 24, 2018, from 2 state cancer registries, social media, and clinician referrals to participate in a study of posttreatment ovarian function. Data analysis occurred between March 1 and September 1, 2022.

**Exposures:**

Participants reported their menstrual pattern. Estimated treatment gonadotoxicity was ascertained through medical record review.

**Main Outcomes and Measures:**

Participants reported infertility risk perception and were categorized as increased risk (feeling less fertile or unable to become pregnant) or no increased risk (feeling more or as fertile) compared with female individuals their age. Objective infertility risk was determined by estimated gonadotoxicity, menstrual pattern, and ovarian reserve testing of self-collected dried blood spots. Multivariable logistic regression identified factors associated with perceived infertility and underestimation or overestimation of infertility risk.

**Results:**

This study included 785 female participants with a mean (SD) age of 33.2 (4.8) years at enrollment and 25.9 (5.7) years at diagnosis. Most participants self-identified their race and ethnicity as White (585 [74.5%]) and non-Hispanic (628 [78.7%]). Most participants (483 [61.5%]) perceived a higher risk of infertility compared with female participants their age. Prior exposure to moderate- or high-gonadotoxicity treatments was associated with higher odds of perceiving increased infertility risk compared with exposure to low-gonadotoxicity treatments (adjusted odds ratio [AOR], 2.73 [95% CI, 1.87-3.97] and 15.39 [95% CI, 5.52-42.96], respectively). Amenorrhea and irregular cycles were associated with higher odds of perceiving increased infertility risk (AOR, 3.98 [95% CI, 2.13-7.41] and 1.69 [95% CI, 1.19-2.40], respectively). Perceived infertility risk had minimal agreement with objective risk (κ = 0.19). Multiparity (AOR, 4.17 [95% CI, 2.61-6.64]) was associated with increased odds of underestimation, while older age (AOR, 0.94 [95% CI, 0.89-0.98]), endocrine comorbidity (AOR, 0.35 [95% CI, 0.18-0.69]), and prior infertility (AOR, 0.16 [95% CI, 0.07-0.38]) were associated with lower odds of underestimation. Multiparity (AOR, 0.48 [95% CI, 0.27-0.86]), breast cancer (AOR, 0.38 [95% CI, 0.20-0.73]), and skin cancer (AOR, 0.24 [95% CI, 0.11-0.51]) were associated with lower odds of overestimation.

**Conclusions and Relevance:**

In this cohort study, survivors of AYA cancer had high rates of perceiving increased infertility risk but frequently overestimated or underestimated their risk. These findings suggest that counseling on infertility risk throughout survivorship may reduce misalignment between perceptions and actual risk, decrease fertility-related psychological distress, and inform family planning decisions.

## Key Points

**Question:**

How do female survivors of adolescent and young adult (AYA) cancer perceive their fertility?

**Findings:**

In this cohort study of 785 participants, most female survivors (62%) of AYA cancer perceived increased risk of infertility, particularly with increased estimated gonadotoxicity of cancer treatment or an abnormal menstrual pattern. However, their perceptions of infertility risk had minimal agreement with objective risk.

**Meaning:**

These findings suggest that infertility risk counseling throughout cancer survivorship is needed for AYA cancer survivors to reduce misalignment between perceptions and actual risk, decrease fertility-related psychological distress, and inform family planning decisions.

## Teaser

This cohort study examines factors associated with infertility risk perception among female survivors of adolescent and young adult cancer and tests the concordance between risk perception and objective infertility risk.

## Introduction

Infertility among female survivors of adolescent and young adult (AYA) cancer can stem from cancer treatment–related depletion of the finite ovarian reserve, disruption of hypothalamic-pituitary-ovarian function, and/or injury to the uterus.^1^ Survivors of AYA cancer experience more infertility and fewer live births compared with siblings without cancer and survivors of childhood cancer.^2,3^ Although many AYA cancer survivors retain the potential to have children after cancer, infertility concerns cause substantial psychological distress, and misperceptions about fertility potential can lead to unplanned pregnancies and/or misinformed reproductive decisions.^4,5,6,7,8,9^

Data on fertility perceptions of AYA cancer survivors and their alignment with objective infertility risk are scant. Prior studies, largely in childhood cancer survivors, report that survivors often overestimate or underestimate their infertility risk.^2,10,11,12^ Lehmann et al^13^ found low agreement between survivors’ perception of their infertility risk and their objective gonadal function. Adult women with chronic conditions including cancer also inaccurately assess the impact of their condition on fertility.^14^ A majority of AYA cancer survivors report infertility concerns,^15,16^ but little is known about factors associated with infertility risk perception and how perceived risk compares with objective risk. Such data could inform which survivors have greater need for fertility counseling and related care.

This study aimed to characterize factors associated with infertility risk perception among female AYA cancer survivors. Because treatment gonadotoxicity has been associated with infertility risk perception among childhood cancer survivors and menstrual pattern has been associated with fertility perception among women,^13,17,18,19^ we hypothesized that increased estimated treatment gonadotoxicity and amenorrhea or an irregular menstrual pattern would be associated with higher infertility risk perception. Second, this study aimed to evaluate the agreement between risk perception and objective risks to fertility, as measured by estimated treatment gonadotoxicity and ovarian reserve markers. We hypothesized that survivors’ infertility risk perception would have poor agreement with objective risk.

## Methods

### Study Design and Participants

The Reproductive Window Study was a prospective cohort study estimating the trajectory of posttreatment ovarian function among female survivors of AYA cancers.^20^ Participants were recruited through the California and Texas cancer registries (386 [36.0%]), the University of California San Diego Health System (317 [29.6%]), social media (326 [30.5%]), and physician referrals (42 [3.9%]). Between March 25, 2015, and September 24, 2018, 1071 individuals were consented to the study and completed the baseline survey. For the current analysis, participants of the parent study who completed baseline surveys, had complete medical record abstraction of oncologic treatments, and did not undergo hysterectomy were included. The State of California Committee for the Protection of Human Subjects and the institutional review boards at the University of California San Diego and the Texas Department of State Health Services approved the study. Participants provided written informed consent. The study followed the Strengthening the Reporting of Observational Studies in Epidemiology (STROBE) reporting guideline.

Eligibility criteria included female sex, age 18 to 39 years, cancer diagnosis between age 15 and 39 years, completion of primary cancer treatment, and at least 1 ovary. Participants could enter the study from 1 day to 25 years posttreatment. The included cancer types were selected by highest incidence in this population: breast, leukemia, lymphoma, gynecologic (cervix, uterus, or ovary), gastrointestinal, sarcomas, skin, and thyroid. Exclusion criteria were uncontrolled endocrinopathies (eg, diabetes or thyroid or adrenal disease), multiple cancers, or recurrence.

Participants were followed for up to 18 months. They were asked to complete an online questionnaire through the study portal and to self-collect dried blood spots (DBSs) at baseline and every 6 months thereafter for 4 total assessments. Questionnaires collected self-reported information on cancer, reproductive (fertility, contraception, and menstrual pattern), medical, demographic, and lifestyle characteristics using questions derived from large cancer and reproductive cohort studies.^21,22^ Participant self-collected DBSs for measuring endocrine biomarkers were shipped to the research team.^20^ Menstruating participants collected DBSs in the early follicular phase (cycle days 3-7); amenorrheic participants collected DBSs on a random day.

Participants consented to medical record information release (under the Health Insurance Portability and Accountability Act Privacy Rule) for study staff to obtain primary cancer treatment records. Participants reported demographics, medical history, reproductive history, and menstrual pattern via an online questionnaire. Cancer diagnosis and treatment data were abstracted by 2 board-certified pediatric oncologists and 1 board-certified reproductive endocrinologist using the Childhood Cancer Survivor Study methods.^23^ High agreement was reported on rereview of 25% of the abstracted data.^20,24^

### Measures

#### Perceived Infertility Risk

Participants were asked about their perceived fertility risk (“How do you feel about your own fertility [ability to get pregnant] right now?”) compared with female individuals their age. Responses included the following: “I feel I am more fertile,” “I think I am as fertile,” “I think I am less fertile,” and “I think I am unable to get pregnant.”^8^ Responses were collapsed to compare any perception of increased risk (less fertile or unable to get pregnant) to no perception of increased risk (included same or more fertile).

#### Estimated Gonadotoxicity of Cancer Treatments

Treatments were categorized into 3 groups: low, moderate, and high gonadotoxicity. Categorization was determined by literature review on toxicity on ovarian reserve.^25,26,27,28,29^ High-toxicity treatments included any pelvic radiation, stem cell or bone marrow transplants (autologous or allogeneic), or a cyclophosphamide equivalent dose of 7 g/m^2^ or greater. Low-toxicity treatments (ie, not expected to be gonadotoxic) included surgery only (excluding oophorectomy), endocrine therapy only, and radioiodine treatment. All remaining exposures were classified as moderate toxicity; these included biologics, platinum agents, combination doxorubicin-bleomycin-vinblastine-dacarbazine, hyperthermic intraperitoneal chemotherapy, unilateral oophorectomy, trachelectomy, cyclophosphamide equivalent dose of less than 7 g/m^2^, and bevacizumab.

#### Menstrual Pattern and Ovarian Reserve Markers as Measures of Ovarian Function

Number of menses in the past year and cycle variations in bleeding pattern were used to categorize menstrual pattern.^24,30^ Participants were grouped as having regular, irregular, or no menstrual cycles. Individuals reporting 10 to 12 menses in the last year without interbleeding intervals of more than 60 days apart were categorized as having regular menstrual cycles.^30^ Those who reported 9 or fewer menses in the past year were categorized as having irregular menstrual cycles. Finally, participants reporting amenorrhea in the past year included those who never experienced menarche.

Dried blood spot collection is valid and reliable for detection of antimüllerian hormone (AMH) and follicle-stimulation hormone (FSH) levels.^31,32^ Both AMH and FSH levels were assessed through enzyme-linked immunosorbent assays designed specifically for DBS samples (limit of detection, 0.03 ng/mL and 0.07 mIU/mL, respectively; and interassay and intraassay coefficient of variation, <10%) (AL-129 and AL-187; Ansh Labs).^20^ To reduce misclassification based on an acute effect of cancer treatment, we excluded AMH and FSH levels collected within 2 years of treatment.^24^ For participants within 2 years of treatment, we used their menstrual pattern only. We excluded FSH levels in female individuals receiving hormonal therapy. An AMH level of less than 1 ng/mL and an FSH level greater than 10 IU/L were considered evidence of impaired fertility.^24,33,34,35,36^

#### Objective Infertility Risk

Participants were categorized as having objective infertility risk (or not) based on estimated treatment gonadotoxicity, receipt of hormone therapy, and measures of ovarian function (eFigure in Supplement 1). Hormone therapy included hormone replacement therapy, hormonal contraceptives, and progestin intrauterine devices. Participants treated with moderate- or high-gonadotoxicity treatments regardless of menstrual pattern and ovarian reserve test results were categorized as having objective infertility risk. In addition, among those exposed to low-gonadotoxicity treatments, individuals with low AMH levels, high FSH levels, hormone replacement therapy receipt, and irregular or no menstrual cycles were categorized as having objective infertility risk. Within the low-gonadotoxicity treatment exposure group, participants receiving hormone therapy and who did not have AMH levels could not be classified.

#### Covariates

Demographic variables were self-reported and included age, self-identified race and ethnicity, sexual orientation, education, income, marital status, and health insurance coverage. Data on race (American Indian or Alaska Native, Asian, or Native Hawaiian or other Pacific Islander; Black; White; or other or multiple races) and ethnicity (Hispanic or non-Hispanic) were obtained because they were hypothesized to be associated with fertility perception. Respondents ranked their overall general health with 5 responses from excellent to poor. Body mass index was calculated as self-reported weight in kilograms divided by self-reported height in meters squared. Self-reported comorbidities were categorized as cardiovascular or pulmonary, endocrine (diabetes or thyroid related), psychological, and other as applicable. Psychosocial factors included stress measured with the 10-item Perceived Stress Scale,^37^ depression measured with the Patient Health Questionnaire depression scale,^38^ and social support measured with the RAND Medical Outcomes Study survey.^39^

### Statistical Analysis

Data analysis occurred between March 1 and September 1, 2022. All analyses were conducted with R Studio, version 1.2.5001 (R Project for Statistical Computing). On factors associated with perceived infertility risk, the exposures of interest were estimated gonadotoxicity of cancer treatments and menstrual pattern. Following descriptive analysis, bivariable analyses estimated associations between exposures and outcomes using χ^2^, Fisher exact, and *t* tests as appropriate. Covariates closely associated with one another (ρ ≥ 0.5) were reduced to include 1 of the 2 variables in the final model. Binomial logistic regression was utilized in multivariable analyses. The model was built as an explanatory model in which all covariates were included and then reduced if nonsignificant in the model and did not present confounding (≤10% change in parameter). We conducted 2-sided hypothesis testing with significance set at *P* < .05.

Accurate perception of infertility risk was assessed by the percent and degree of agreement with objective infertility risk. The Cohen κ statistic was used to evaluate the degree of agreement between measures. Agreement between objective infertility risk and perceived infertility risk was classified as accurate, an underestimate of risk, or an overestimate of risk. Characteristics associated with risk assessment were assessed via multinomial regression models. Per an explanatory model process, all covariates identified as potentially associated were included and then reduced if nonsignificant and did not present confounding.

## Results

This analysis included 785 of the 1071 participants in the parent study (Table 1). Their mean (SD) age at diagnosis was 25.9 (5.7) years, and their mean age at study enrollment was 33.2 (4.8) years. A total of 54 participants (6.9%) self-identified as American Indian or Alaska Native, Asian, or Native Hawaiian or other Pacific Islander, 23 (2.9%) as Black, 585 (74.5%) as White, and 108 (13.8%) as other or multiple races. Participants reported their ethnicity as Hispanic (167 [21.3%]) or non-Hispanic (628 [78.7%]).

**Table 1.** Demographic, Cancer, and Fertility Characteristics of Female Adolescent and Young Adult Cancer Survivors by Perceived Infertility Risk^a^

Characteristic	Total sample (N = 785)	Perceived infertility risk		*P* value
		Not increased (n = 302)	Increased (n = 483)	
Age, mean (SD), y				
At enrollment	33.2 (4.8)	33.6 (4.6)	33.0 (4.9)	.13
At diagnosis	25.9 (5.7)	25.4 (5.6)	26.2 (5.7)	.06
Race				
American Indian or Alaska Native, Asian, or Native Hawaiian or other Pacific Islander	54 (6.9)	21 (7.0)	33 (6.8)	.48
Black	23 (2.9)	8 (2.6)	15 (3.1)	
White	585 (74.5)	230 (76.2)	355 (73.5)	
Other or multiple races^b^	108 (13.8)	34 (11.3)	74 (15.3)	
Ethnicity				
Hispanic	167 (21.3)	60 (19.9)	107 (22.2)	.53
Non-Hispanic	628 (78.7)	242 (80.1)	376 (77.8)	
Sexual orientation				
Heterosexual	735 (93.6)	283 (93.7)	452 (93.6)	>.99
Other	50 (6.4)	19 (6.3)	31 (6.4)	
Relationship status				
Married or living together	546 (69.6)	218 (72.2)	328 (67.9)	.21
Not married or living together	239 (30.4)	84 (27.8)	155 (32.1)	
College education or higher				
Yes	605 (77.1)	241 (79.8)	364 (75.4)	.18
No	180 (22.9)	61 (20.2)	119 (24.6)	
Employment status				
Employed	609 (77.6)	242 (80.1)	367 (76.0)	.34
Not employed	176 (22.4)	60 (19.9)	116 (24.0)	
Annua household income, $				
<51 000	234 (29.8)	70 (23.2)	164 (34.0)	.003^c^
≥51 000	551 (70.2)	232 (76.8)	319 (66.0)	
Health insurance status				
Yes	760 (96.8)	295 (97.7)	465 (96.3)	.38
No	25 (3.2)	7 (2.3)	18 (3.7)	
BMI				
<18.5	34 (3.1)	10 (3.3)	14 (2.9)	.63
18.5-24.9	363 (46.2)	135 (44.7)	228 (47.2)	
25-29.9	178 (22.7)	75 (24.8)	103 (21.3)	
≥30	198 (25.2)	72 (23.8)	126 (26.1)	
General health				
Excellent	82 (10.4)	37 (12.3)	45 (9.3)	.01^c^
Very good	325 (41.4)	134 (44.4)	191 (39.5)	
Good	296 (37.7)	112 (37.1)	184 (38.1)	
Fair or poor	80 (10.2)	18 (6.0)	62 (12.8)	
Comorbidity				
Cardiovascular or pulmonary	115 (14.6)	35 (11.6)	80 (16.6)	.07
Endocrine	148 (18.9)	43 (14.2)	105 (21.7)	.01^c^
Psychological	204 (26.0)	66 (21.9)	138 (28.6)	.04^c^
Other	265 (33.8)	104 (34.4)	161 (33.3)	.81
Stress				
None or low	313 (39.9)	135 (44.7)	178 (36.9)	.003^c^
Moderate	423 (53.9)	158 (52.3)	265 (54.9)	
High	49 (6.2)	9 (3.0)	40 (8.3)	
Depression				
None	226 (28.8)	76 (25.2)	150 (31.1)	.22
Mild	105 (13.4)	35 (11.6)	70 (14.5)	
Moderate	42 (5.4)	13 (4.3)	29 (6.0)	
Moderately severe or severe	12 (1.5)	3 (1.0)	9 (1.9)	
Social support, mean (SD)^d^	4.3 (0.8)	4.3 (0.8)	4.3 (0.8)	.51
Cancer type				
Thyroid	154 (19.6)	77 (25.5)	77 (15.9)	<.001^c^
Breast	209 (26.6)	71 (23.5)	138 (28.6)	
Blood, leukemia, or lymphoma	268 (34.1)	93 (30.8)	175 (36.2)	
Reproductive (cervix, uterus, ovary)	58 (7.4)	17 (5.6)	41 (8.5)	
Gastrointestinal	23 (2.9)	7 (2.3)	16 (3.3)	
Bone or soft tissue	49 (6.2)	19 (6.3)	30 (6.2)	
Skin	24 (3.1)	18 (6.0)	6 (1.2)	
Time since treatment completion, y				
0-2	44 (5.6)	12 (4.0)	32 (6.6)	.01^c^
3-4	140 (17.8)	45 (14.9)	95 (19.7)	
5-8	326 (41.5)	119 (39.4)	207 (42.9)	
≥9	275 (35.0)	126 (41.7)	149 (3.8)	
Estimated treatment gonadotoxicity				
Low	225 (28.7)	125 (41.4)	100 (2.7)	<.001^c^
Moderate	479 (61.0)	173 (57.3)	306 (63.4)	
High	81 (10.3)	4 (1.3)	77 (15.9)	
Menstrual pattern				
Regular periods	316 (40.3)	158 (52.3)	158 (32.7)	<.001^c^
Irregular periods	360 (45.9)	125 (41.4)	235 (48.7)	
Amenorrhea	109 (13.9)	19 (6.3)	90 (18.6)	
Parity				
0	488 (62.2)	139 (46.0)	349 (72.3)	<.001^c^
≥1	297 (37.8)	163 (54.0)	134 (27.7)	
Ever visited fertility specialist				
Yes	248 (31.6)	60 (19.9)	188 (38.9)	<.001^c^
No	537 (68.4)	242 (80.1)	295 (61.1)	
Ever received fertility treatment				
Yes	149 (19.0)	32 (10.6)	117 (24.2)	.28
No	636 (81.0)	270 (89.4)	366 (75.8)	
Previous fertility preservation (oocyte, embryo, or ovarian tissue banking)				
Yes	57 (7.3)	13 (4.3)	44 (9.1)	.45
No	728 (92.7)	289 (95.7)	439 (90.9)	
Prior infertility				
Yes	108 (13.8)	15 (5.0)	93 (19.3)	<.001^c^
No	677 (86.2)	287 (95.0)	390 (80.7)	
Hormone therapy over last 12 mo				
Yes	356 (45.4)	110 (36.4)	246 (5.9)	<.001^c^
No	429 (54.6)	192 (63.6)	237 (49.1)	

Abbreviation: BMI, body mass index (calculated as weight in kilograms divided by height in meters squared).

^a^ Variables depicted as No. (%) unless otherwise indicated.

^b^ Participants who self-identified as multiple or other races.

^c^ *P* < .05.

^d^ Support was captured in 4 domains: emotional/informational, tangible, affectionate, and postive social interaction.

The excluded participants were more likely to be Hispanic, to be parous, and to report worse general health; they were less likely to have completed college and to have breast cancer. Sixteen participants were excluded due to hysterectomy.

Most included participants were married or living with their partner (546 [69.6%]) and had completed college (605 [77.1%]). The most common cancer types were blood cancers (leukemia or lymphoma; 268 [34.1%]), breast (209 [26.6%]), and thyroid (154 [19.6%]). Categorized by cancer treatment exposure, 225 (28.7%), 479 (61.0%), and 81 (10.3%) participants received low-, moderate-, and high-gonadotoxicity treatments, respectively. A total of 316 participants (40.3%) reported a regular menstrual pattern, 360 (45.9%) reported irregular menstrual cycles, and 109 (13.9%) were amenorrheic. Of the 109 participants who were amenorrheic, 23 (21.1%) used hormonal contraception or had a progestin intrauterine device. Most participants (483 [61.5%]) perceived an increased risk of infertility. A description of participant characteristics by estimated treatment gonadotoxicity is presented in the eTable in Supplement 1.

### Estimated Treatment Gonadotoxicity, Menstrual Pattern, and Perceived Infertility Risk

In bivariable analysis, estimated treatment gonadotoxicity and menstrual pattern were associated with perceived infertility risk (Table 1). Comorbidities, cancer type and proximity to treatment, and reproductive characteristics were also associated with perception of infertility risk.

Due to multicollinearity, age at enrollment and stress were retained in all multivariable models, whereas age at diagnosis and depression were removed. In adjusted analysis, moderate- and high-gonadotoxicity treatments were associated with increased perceived infertility risk (Table 2). Compared with participants exposed to low-gonadotoxicity treatments, those exposed to moderate-gonadotoxicity treatments had 2.73-fold higher odds (95% CI, 1.87-3.97) of perceiving increased infertility, whereas those exposed to high-gonadotoxicity treatments had 15.39-fold higher odds (95% CI, 5.52-42.96). Amenorrhea and irregular menstrual cycles were also associated with increased infertility risk perception (adjusted odds ratio [AOR], 3.98 [95% CI, 2.13-7.41] and 1.69 [95% CI, 1.19-2.40], respectively) (Table 2). In addition, endocrine comorbidity and prior infertility were associated with increased perception of infertility risk, whereas having prior births was protective.

**Table 2.** Unadjusted and Adjusted Models of the Characteristics Associated With Perceived Infertility Risk

Characteristic	Perceived infertility risk, OR (95% CI)	
	Unadjusted	Adjusted
Estimated treatment gonadotoxicity		
Low	1 [Reference]	1 [Reference]
Moderate	2.18 (1.58-3.01)	2.73 (1.87-3.97)
High	21.59 (8.09-54.62)	15.39 (5.52-42.96)
Menstrual pattern		
Regular	1 [Reference]	1 [Reference]
Amenorrhea	2.73 (1.55-4.79)	3.98 (2.13-7.41)
Irregular	1.69 (1.23-2.31)	1.69 (1.19-2.40)
Endocrine comorbidities	2.24 (1.47-3.41)	2.30 (1.46-3.63)
Parity		
0	1 [Reference]	1 [Reference]
≥1	0.33 (0.24-0.45)	0.21 (0.14-0.30)
Prior infertility	4.76 (2.69-8.43)	8.46 (4.54-15.75)

Abbreviation: OR, odds ratio.

### Agreement Between Objective and Perceived Infertility Risk

A total of 475 participants (60.5%) were categorized as having objective infertility risk, whereas 179 (22.8%) were categorized as not having objective infertility risk. There were 131 participants (16.6%) who could not be categorized due to lack of ovarian reserve testing while receiving hormonal therapy. Overall, of the 654 participants, 417 (63.8%) accurately assessed their risk (96 [14.7%] had no objective or perceived increased risk, and 321 [49.1%] had objective and perceived increased risk), 83 (12.7%) overestimated their risk, and 154 (23.5%) underestimated their risk. Perceived infertility risk had minimal agreement (κ = 0.19) with objective risk.

In adjusted analysis, participants who were older (AOR, 0.94 [95% CI, 0.89-0.98]) or had endocrine comorbidity (AOR, 0.35 [95% CI, 0.18-0.69]) and who had prior infertility (AOR, 0.16 [95% CI, 0.07-0.38]) or gastrointestional cancer (AOR, 0.35 [95% CI, 0.13-0.91]) were less likely to underestimate infertility risk, whereas prior birth was associated with higher odds of underestimation (AOR, 4.17 [95% CI, 2.61-6.64]) (Table 3). Accordingly, those with prior birth were less likely to overestimate their risk. Compared with thyroid cancer survivors, breast (AOR, 0.38 [95% CI, 0.20-0.73]) and skin (AOR, 0.24 [95% CI, 0.11-0.51]) cancer survivors were less likely to overestimate their risk.

**Table 3.** Adjusted Models of Characteristics Associated With Underestimation and Overestimation of Perceived Infertility Risk

Characteristic	Accurate estimation (n = 417), No. (%)	Underestimation (n = 154)		Overestimation (n = 83)	
		No. (%)	AOR (95% CI)	No. (%)	AOR (95% CI)
Age at enrollment, mean (SD), y	33.5 (4.8)	33.3 (4.8)	0.94 (0.89-0.98)^a^	33.9 (4.5)	1.04 (1.00-1.11)
Parity					
0	262 (62.8)	63 (40.9)	1 [Reference]	60 (72.3)	1 [Reference]
≥1	155 (37.2)	91 (59.1)	4.17 (2.61-6.64)^a^	23 (27.7)	0.48 (0.27-0.86)^a^
Endocrine comorbidities	78 (18.7)	13 (8.4)	0.35 (0.18-0.69)^a^	23 (27.7)	1.22 (0.68-2.20)
Cancer type					
Thyroid	55 (13.2)	28 (18.2)	NA	25 (30.1)	NA
Breast	126 (30.2)	47 (30.5)	0.59 (0.33-1.08)	14 (16.9)	0.38 (0.20-0.73)^a^
Blood, leukemia, or lymphoma	153 (36.7)	54 (35.1)	0.37 (0.4-1.00)	27 (32.5)	0.66 (0.27-1.62)
Reproductive (cervix, uterus, ovary)	31 (7.4)	7 (4.5)	0.33 (0.08-1.36)	9 (10.8)	0.40 (0.08-1.96)
Gastrointestinal	12 (2.9)	3 (1.9)	0.35 (0.13-0.91)^a^	2 (2.4)	0.32 (0.10-1.04)
Bone or soft tissue	31 (7.4)	8 (5.2)	1.26 (0.38-4.16)	4 (4.8)	0.46 (0.09-2.35)
Skin	9 (2.2)	7 (4.5)	0.63 (0.34-1.19)	2 (2.4)	0.24 (0.11-0.51)^a^
Prior infertility	74 (17.7)	7 (4.5)	0.16 (0.07-0.38)^a^	14 (16.9)	1.01 (0.51-1.97)

Abbreviations: AOR, adjusted odds ratio; NA, not applicable.

^a^ *P* < .05.

## Discussion

This study characterized factors associated with fertility perception and estimated the accuracy of perceived infertility risk among female AYA cancer survivors. Studying risk perceptions directly is of interest because they are precursors to health behaviors, including information seeking and medical care compliance^40^; they are distinct from measures of reproductive concerns,^41,42^ which only measure a component of risk perception.^43,44^ Both estimated gonadotoxicity of cancer treatment and absent or irregular menses were associated with higher infertility risk perception. The poor agreement observed between infertility risk perception and objectively measured risk highlights a critical gap in survivorship knowledge. In the context of AYA cancer survivorship, the identification of these populations who perceive increased infertility risk or inaccurately perceive risk contributes to clinically tailoring communication, fertility surveillance, and fertility treatments and family planning decision making.

As hypothesized, moderate and high estimated gonadotoxicity treatments were associated with higher odds of increased infertility risk perceptions, similar to observations among childhood cancer survivors.^13^ The mechanism by which the estimated gonadotoxicity of cancer treatment affects risk perception may be through more frequent counseling of patients who faced more gonadotoxic treatments. Although we unfortunately do not have data from this cohort on whether they had been counseled on their fertility risks, separate cohorts of young cancer survivors demonstrate that more counseling is observed when patients undergo chemotherapy or radiation compared with surgery alone.^15,45^ Importantly, more specific treatments (eg, chemotherapy regimen, targeted therapy, and radiation dose) are difficult to recall,^12,13,46^ and fertility risks differ by treatments. Thus, treatment summaries and repeat counseling on outcomes associated with acute or long-term effects of specific treatments on fertility are necessary components of quality survivorship care.^47^

Amenorrhea and irregular bleeding patterns are associated with infertility risk perception because menstrual pattern is part of normal bodily function and can be self-monitored by AYA cancer survivors.^48^ Biologically, menstrual pattern reflects ovarian function, and detection of abnormal patterns can contribute to detection of reproductive aging, specifically entry into the menopausal transition, in the general population.^17,18,19,34^ Any irregularity (even when using contraceptives) is cause for concern in the general population.^17,48,49,50^ Posttreatment menstrual pattern, in the absence of exogenous sex steroid hormones (eg, hormonal contraception) and/or endocrine therapy (eg, tamoxifen or gonadotropin hormone-releasing hormone agonists), can be used to assess ovarian function, which is required for fertility. Survivors and their clinicians need to be aware that menstrual pattern alone may not be a reliable indicator of residual ovarian reserve, particularly within the first 2 to 3 years post treatment during ovarian function recovery.^24^

Ovarian reserve measures were additive to treatment exposure and menstrual pattern in defining objective risk. A substantial proportion of participants were receiving exogenous hormones, and menstrual pattern would not reflect ovarian reserve. Antimüllerian hormone allows for measurement of ovarian reserve because it is consistent throughout menstrual cycles even when patients are taking hormonal contraception, whereas FSH can fluctuate.^51^ For cancer survivors aged 25 years or older who have undergone gonadotoxic treatment, AMH in conjunction with FSH is recommended to identify premature ovarian insufficiency.^52^ For individuals with AYA in particular, AMH and FSH, combined with menstrual pattern, provide a more accurate assessment of ovarian reserve than menstrual pattern alone.^24,33^

This study observed minimal agreement between perceptions of infertility risk compared with objective risks. Although 63.8% of participants (417 of 654) accurately assessed their risk, the Cohen κ requires a rate of 80% or greater to qualify as high agreement.^53^ A similar κ value (κ = 0.19) was found in a study among survivors of childhood cancer between perceived and gonadal function–defined fertility risk.^13^ In contrast, AYA-aged survivors of childhood cancer had moderate agreement (κ = 0.66) between FSH levels and self-reported premature ovarian insufficiency; repeated interactions with pediatric cancer survivorship clinics or endocrinologists were associated with high agreement (κ = 0.83 and κ = 1.0, respectively).^10^ Higher agreement may be because the premature ovarian insufficiency phenotype of prolonged amenorrhea is known to the patient or because participants received appropriate survivorship care. We observed that participants with better reproductive outcomes (parous, not infertile) were more likely to underestimate infertility risk. We speculate that survivors of cancers like breast cancer may have been more likely to be accurate because fertility risk counseling may be more frequent in some cancers, but these data by cancer type are exploratory and require future validation.^54^ Because a substantial proportion of our participants either overestimated or underestimated their infertility risk, these prior studies indicate a need to provide reproductive counseling to AYA cancer survivors. An added challenge is that AYA cancer survivors are initially seen and later followed in more varied clinical settings than childhood cancer survivors, lending to less fidelity with routine reproductive counseling at diagnosis and throughout survivorship.^55,56,57^

### Strengths and Limitations

A strength of this study is its use of 3 key objective measures of infertility risk: estimated cancer treatment gonadotoxicity, menstrual pattern, and ovarian reserve markers. Using AMH and FSH in combination with hormonal therapy and menstrual pattern allowed for better estimation of ovarian function in this study and follows expert recommendations for AYA cancer survivors.^24,58^ Other studies mainly used treatment gonadotoxicity to estimate impaired fertility and only a few included biomarkers, most often limited to FSH.^10,11,12^ Another strength was a sufficient population to assess associations with both under- and overestimation of risk.

A limitation of this study is that there is not a standardized risk stratification, and it is possible that different data or consensus statements used by providers could change categorization of risk, consequently affect the counseling provided to survivors, and alter our estimates of agreement between objective and perceived risk. Another limitation is the generalizability of these findings, as participants were recruited to engage in online and remote data collection to a study on reproductive health after cancer. We do not know whether AYA cancer survivors who chose not to participate in the study had fewer resources or less health literacy, thus we may have underestimated the proportion with misaligned risk perception.

## Conclusions

As the findings of this cohort study suggest, fertility information following cancer treatment continues to be an unmet need for AYA cancer survivors.^59,60^ Age-appropriate and repeated fertility counseling throughout survivorship care informed by objective risk measures may help reduce concerns and misperceptions around fertility. Key groups of survivors identified in this study may be particularly susceptible to psychological burden and distress. Past studies with AYA cancer survivors show that the use of survivorship care plans can improve infertility concerns and reduce unmet information needs.^61,62^ Strategies to reduce misalignment between perceptions and actual risk are essential to reducing psychological distress and allow for better informed reproductive decisions for AYA cancer survivors.

## Notes

Supplement 1. **eFigure.** Categorization of Objective Infertility Risk Among Study Participants **eTable.** Risk Perception, Demographic, Cancer, and Fertility Characteristics of Female Survivors of Adolescent and Young Adult Cancer by Estimated Treatment Gonadotoxicity

Supplement 2.
Data Sharing Statement
