# Supplementary material for: Perceived and Objective Fertility Risk Among Female Survivors of Adolescent and Young Adult Cancer
Source: JAMA Netw Open. 2023 Oct 11;6(10):e2337245. doi: 10.1001/jamanetworkopen.2023.37245 (PMC10568355; doi:10.1001/jamanetworkopen.2023.37245)
Supplement: Supplement 1. — eFigure. Categorization of Objective Infertility Risk Among Study Participants eTable. Risk Perception, Demographic, Cancer, and Fertility Characteristics of Female Survivors of Adolescent and Young Adult Cancer by Estimated Treatment Gonadotoxicity [file jamanetwopen-e2337245-s001.pdf]

Supplementary Online Content

Din HN, Singh-Carlson S, Corliss HL, et al. Perceived and objective fertility risk among female survivors of adolescent and young adult cancer. *JAMA Netw Open*. 2023;6(10):e2337245. doi:10.1001/jamanetworkopen.2023.37245

eFigure. Categorization of Objective Infertility Risk Among Study Participants

eTable. Risk Perception**,** Demographic, Cancer, and Fertility Characteristics of Female Survivors of Adolescent and Young Adult Cancer by Estimated Treatment Gonadotoxicity

This supplementary material has been provided by the authors to give readers additional information about their work.

© 2023 Din HN et al. *JAMA Network Open*.

eFigure. Categorization of Objective Infertility Risk Among Study Participants

A: participants with moderate and high estimated gonadotoxic risk treatments were categorized as having objective infertility risk **B:** Participants with low estimated gonadotoxic treatments were categorized further by receipt and type of hormonal therapy. **C:** Among participants receiving hormonal therapies (not including menopausal therapies) objective fertility risk was determined by results of ovarian reserve testing (ORT). ORT included assessment of AMH and FSH, FSH was only included if a valid test was reported (i.e., participant was not pregnant at time of test). If no ORT was reported in this sub-group, participants were excluded because gonadal functioning could not be determined. **D:** Among participants not receiving any hormonal therapies, menstrual patterns were incorporated into fertility determination along with ORT results as applicable. Again, FSH was only evaluated if it represented a valid test.

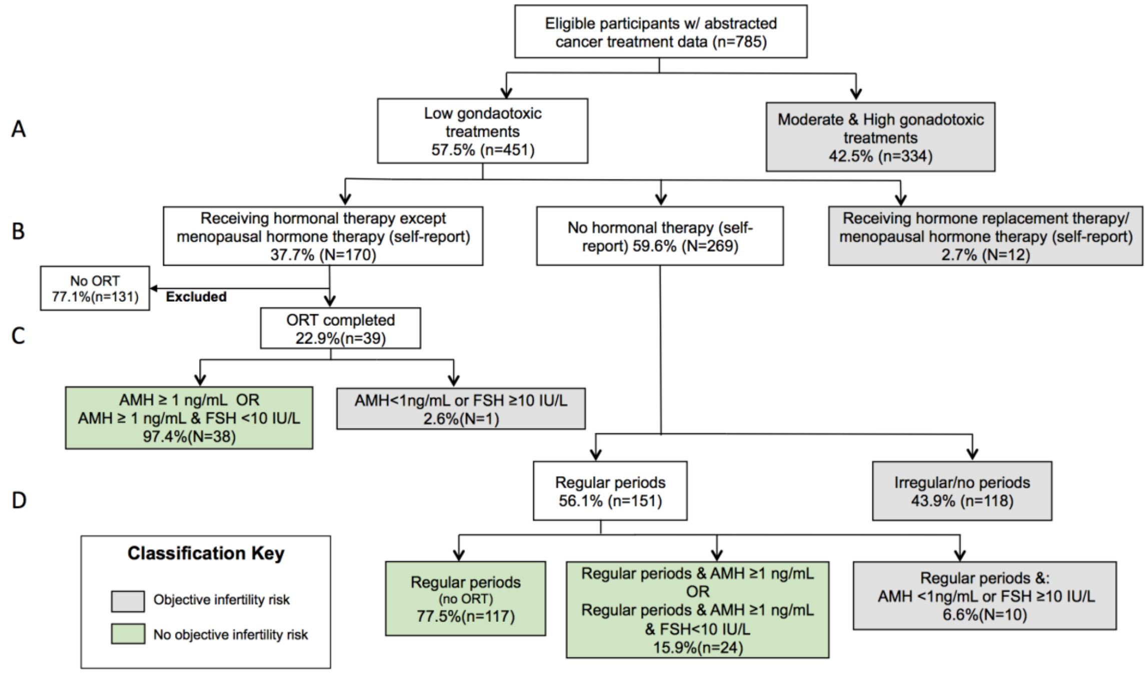

© 2023 Din HN et al. *JAMA Network Open*.

eTable. Risk Perception**,** Demographic, Cancer, and Fertility Characteristics of Female Survivors of Adolescent and Young Adult Cancer by Estimated Treatment Gonadotoxicity[1]

		**Estimated Treatment Gonadotoxicity**			
	Total Sample n=785	Low n=225	Moderat e n=479	High0 n=81	p value
**Perceived Infertility Risk-** Not Increased	302 (38.5)	125 (55.6)	173 (36.1)	4 (4.9)	**<0.001**
Increased	483 (61.5)	100 (44.4)	396 (63.9)	77 (95.1)	
**Menstrual Pattern-** Regular Periods	316 (40.3)	115 (51.1)	190 (39.7)	11 (13.6)	<0.001
Amenorrhea	109 (13.9)	12 (5.3)	58(12.1)	39 (48.1)	
Irregular Periods	360 (45.9)	98 (43.6)	231 (48.2)	31 (38.3)	
**Age at enrollment** (mean (SD))	33.2 (4.8)	33.7 (4.9)	33.1 (4.7)	32.4 (4.9)	**0.04**
**Age at diagnosis** (mean (SD))	25.9 (5.7)	26.0 (5.6)	26.1 (5.8)	24.5 (5.2)	**0.03**
**Race**- Asian/Native Hawaiian/Native Alaskan/ Native Indian	54 (6.9)	18 (8.0)	28 (5.8)	8 (9.9)	.10
Black	23 (2.9)	2 (0.9)	20 (4.2)	1 (1.2)	
White	585 (74.5)	164 (72.9)	363 (75.8)	58 (71.6)	
Mixed/Other race[2]	108 (13.8)	36 (16.0)	60 (12.5)	12 (14.8)	
**Hispanic Ethnicity**	167 (21.3)	50 (22.2)	100 (20.9)	17 (21.0)	0.87
**Heterosexual**	735 (93.6)	207 (92.0)	449 (93.7)	79 (97.5)	0.28
**Relationship status: married/ living together**	546 (69.9)	152 (67.6)	343 (71.6)	51 (63.0)	0.22
**College education**	605 (77.1)	177 (78.7)	367 (76.6)	61 (75.3)	0.77
**Employed**	609 (77.6)	190 (84.4)	366 (76.4)	53 (65.4)	**0.003**
**Household Income ≥$51,000**	551 (70.2)	163 (72.4)	341 (71.2)	47 (58.0)	0.15
**Has health insurance**	760 (96.8)	217 (96.4)	463 (96.7)	80 (98.8)	0.56
**BMI**- <18.5	34 (3.1)	8 (3.6)	14 (2.9)	2 (2.5)	0.14
18.5-24.9	363 (46.2)	103 (45.8)	214 (44.7)	46 (56.8)	
25-29.9	178 (22.7)	43 (19.1)	123 (25.7)	12 (14.8)	
≥30	198 (25.2)	65 (28.9)	115 (24.0)	18 (22.2)	
**General Health-** Excellent	82 (10.4)	21 (9.3)	53 (11.1)	8 (9.9)	0.32
Very Good	325 (41.4)	101 (44.9)	197 (41.1)	27 (33.3)	
Good	296 (37.7)	77 (34.2)	186 (38.8)	33 (40.7)	
Fair/ Poor	80 (10.2)	24 (10.7)	43 (9.0)	13 (16.0)	
**Cardiovascular/Pulmonary Comorbidities**	115 (14.6)	34 (15.1)	62 (12.9)	19 (23.5)	**0.04**
**Endocrine Comorbidities**	148 (18.9)	71 (31.6)	61 (12.7)	16 (19.8)	**<0.001**
**Psychological Comorbidities**	204 (26.0)	56 (24.6)	127 (26.5)	21 (25.9)	0.90
**Other Comorbidities**	265 (33.8)	73 (32.4)	158 (33.0)	34 (42.0)	0.25
**Stress**- No/low stress	313 (39.9)	79 (35.1)	198 (41.3)	36 (44.4)	0.16
Moderate stress	423 (53.9)	134 (59.6)	252 (52.6)	37 (45.7)	
High stress	49 (6.2)	12 (5.3)	29 (6.1)	8 (9.9)	
**Depression**- None	226 (28.8)	63 (28.0)	132 (27.6)	31 (38.3)	0.09
Mild	105 (13.4)	29 (12.9)	68 (14.2)	8 (9.9)	
Moderate	42 (5.4)	19 (8.4)	21 (4.4)	2 (2.5)	
Moderately severe/ Severe	12 (1.5)	5 (2.2)	7 (1.5)	0 (0)	
**Social Support** (mean (SD))	4.3 (0.8)	4.2 (0.9)	4.3 (0.8)	4.3 (0.9)	0.48
**Cancer Type-** Thyroid	154 (19.6)	149 (66.2)	4 (0.8)	1 (1.2)	**<0.001**
Breast	209 (26.6)	0 (0)	209 (43.6)	59 (72.8)	

© 2023 Din HN et al. *JAMA Network Open*.

Blood/ Leukemia/Lymphoma	268 (34.1)	7 (3.1)	43 (9.0)	8 (9.9)	
Reproductive (cervix, uterus, ovary)	58 (7.4)	7 (3.1)	10 (2.1)	6 (7.4)	
Gastrointestinal	23 (2.9)	13 (5.8)	29 (6.1)	7 (8.6)	
Bone/ Soft tissue	49 (6.2)	22 (9.8)	2 (0.4)	0 (0)	
Skin	24 (3.1)	27 (12.0)	182 (38.0)	0 (0)	
**Years since treatment completion-** 0-2 years	44 (5.6)	7 (3.1)	31 (6.5)	6 (7.4)	0.07
3-4 years	140 (17.8)	32 (14.2)	96(20.0)	12 (14.8)	
5-8 years	326 (41.5)	98 (43.6)	199 (41.5)	29 (35.8)	
≥ 9 years	275 (35.0)	88 (39.1)	153 (31.9)	34 (42.0)	
**Parity ≥ 1**	297 (37.8)	90 (40.0)	187 (39.0)	20 (24.7)	**0.03**
**Ever visited fertility specialist**	248 (31.5)	29 (12.9)	179 (37.4)	40 (49.4)	**<0.001**
**Ever received fertility treatment**	149 (60.1)	21 (9.3)	103 (21.5)	25 (30.9)	0.30
**Previous fertility preservation (oocyte, embryo or ovarian tissue banking)**	57 (23%)	6 (2.7)	42 (8.8)	9 (11.1)	0.44
**Previous infertility**	108 (13.8)	31 (13.8)	63 (13.2)	14 (17.3)	0.61
**Hormone therapy over last 12 months**	356 (45.4)	133 (59.1)	258 (53.9)	38 (46.9)	0.14

[1] Variables depicted as n (%) unless otherwise indicated
[2] Other race indicates participants who self-identified as mixed race or some other race

© 2023 Din HN et al. *JAMA Network Open*.
