# Supplementary material for: Perceived and Objective Fertility Risk Among Female Survivors of Adolescent and Young Adult Cancer
Source: JAMA Netw Open. 2023 Oct 11;6(10):e2337245. doi: 10.1001/jamanetworkopen.2023.37245 (PMC10568355; doi:10.1001/jamanetworkopen.2023.37245)
Supplement: Supplement 2. — Data Sharing Statement [file jamanetwopen-e2337245-s002.pdf]

Data Sharing Statement

Din. Perceived and Objective Fertility Risk Among Female Survivors of Adolescent and Young Adult Cancer. *JAMA Netw Open*. Published October 11, 2023.
doi:10.1001/jamanetworkopen.2023.37245

Data
Data available: Yes
Data types: Deidentified participant data, Data dictionary
How to access data: hisu@health.ucsd.edu
When available: With publication

Supporting Documents
Document types: None

Additional Information
Who can access the data: Researchers whose proposed use of the data has been approved.
Types of analyses: For a specified purpose.
Mechanisms of data availability: After approval of a proposal and signed data use agreement.
